# An Answer to Dr. Kinglake, Shewing the Danger of His Cooling Treatment of the Gout

**Published:** 1816-10

**Authors:** 


*An Answer to Dr. Kinglake, shewing the Danger of his cooling Treatment of the Gout.* By JOHN RING, Member of the Royal College of Surgeons, &c. 8vo. pp. 166. London, 1816.

IN common with nine-tenths of the Profession, we have long been so disgusted with the lucubrations of Doctor Kinglake, his satellites, and, we are sorry to say, with some of his antagonists, that the very vehicle in which they are conveyed, has become an object of scorn, pity, and contempt! We appeal to the enlightened, the liberal, the zealous; in short, to the truly estimable portion of Medical Society, whether they do not feel a congeniality of sentiment on this point with ourselves; while by those, who can participate in the disgraceful scenes of contention and abuse with which our periodical Journals have too long abounded, we hope to be execrated in return. Their censure is applause!

Whatever contempt we may feel for the senseless ravings of Dr. Kinglake, and however true may be the assertion of Mr. Ring, that "every one who has the least smattering of medical science loaths them, and no one can relish or digest them but those who can relish and digest every sort of trash:" yet we are far from commending the intemperate language, the acrid sarcasms, which our author has employed in his censures on Doctor Kinglake. Where it is necessary to expose ignorance, to repress arrogance, to humble pride, or to punish delinquency, we are advocates for the lash of indignant but manly satire, and for the poignant arrows of wit, or even ridicule. These are the weapons which we would prefer to the mangling axe or Herculean club, with which Mr. Ring has broken his antagonist on the wheel of controversy. The pages of the *Medico-Chirurgical Journal and Review* shall never be sullied with disgraceful, almost maniacal contests; there are other theatres for the exhibition of such scenes, where an arena is ever open for the Medical Gladiator to vie with the most ferocious of the brute creation! where Billingsgate declamation would be considered Ciceronian eloquence; and where obloquy, scurrility, slander, ribaldry, personal abuse, envy, jealousy, hatred, and every unworthy passion that degrades the human mind, form the scenery of this medical pandemonium! From hence the apple of discord is launched in every direction; and the virtuous, the wise, and the unwary are occasionally *entrapped*, to mingle in this vortex of contention.

This scene of literary prostitution has, at length, like most human evils, worked its own correction and downfall. The more dignified members of the profession have at last seen the necessity of withdrawing from all participation in such graceless impositions on the pockets, and outrages on the feelings of the medical community, by forming themselves into a society for the diffusion of knowledge, unaccompanied by acrid and disgusting controversy. The effect of this measure will be, that those who preferred *garbage* when wholesome food was abundant, will now be left to starve and die on the offal of the medical world.

It requires no prophetic power to predict, that the *Medico-Chirurgical Transactions* will, in a few years, leave those medical journals that depend on original communications for their existence, so scanty a supply as must render them of no value, or force them to adopt other means of subsistence.

From this digression, into which we were led by the controversial volume before us, we return to our analytical labours. We shall confine ourselves, however, entirely to the facts or testimonies which Mr. Ring has brought forward in hostility to the practice, or at least, to the doctrine of Dr. Kinglake in gout; and we believe, that had our author been more industrious in collecting facts, and more sparing of general censure, his work would have been more profitable to his readers, and honourable to himself.

We are of opinion that the warm and stimulating treatment is now banished from the bed-side of gout, as well as of most other diseases, by the generality of practitioners; but that the other extreme, namely, topical applications of cold water, snow, &c. is considered and proved to be equally rash and dangerous in nine cases out of ten.

Incidit in Scyllam cupiens vitare Charybdin!

We shall now state the living evidences which Mr. Ring brings forward, without taking notice of those published documents which were previously before the reader.

" Dr. Curry is so decidedly hostile to the practice, that he has expressed his disapprobation of it in his Lectures for the last fifteen years; and is meditating remarks on the subject, to be published in the Transactions of one of the most respectable Societies in this metropolis. He considers every instance adduced, of the success of Dr. Kinglake's cooling treatment, as only a fortunate escape.

" His opinion is the result of considerable reading, and twenty-five years attentive observation of the disease. He observes, that the danger of sudden and fatal translation of the disorder to the stomach, brain, heart, diaphragm, or other parts, from the repelling of gouty inflammation, is one which has been ascertained by many more examples than have ever reached the public ear.

" He himself has long been a great sufferer from the gout; and has had personal experience of what he emphatically calls, that infernal article the *Eau Medicinale;* by twice taking of which, in small quantities, under circumstances of peculiar urgency and impatience, he has undergone unutterable torments for eighteen months; and is now only rallying his strength and spirits, in consequence of a good constitution, and a regular fit of the disease."

" Dr. Baillie informs me, that he has never recommended cold applications to any joint affected with the gout; but has occasionally heard of the ill effects of that practice in his intercourse with medical men.

" Sir Gilbert Blane also informs me, that he has had no personal experience of the effects of cold water, externally applied, in the gout; but has received such testimony of its ill effects from others, as led him to think it an unsafe remedy."

Mr. Kinder, 12, North Place, Gray's Inn Lane, had the gout in his foot. On the application of cold water and vinegar to the part affected, the pain left his foot and attacked his head with great violence for eight or ten hours, when it returned to the part originally affected.

Dr. Gower informs Mr. Ring, that he has known two instances of the ill effects of cold water externally applied in the gout. In both cases, the head was so much affected that there was a total deprivation of sense and of sight for several hours. It was only by means of powerful stimulants, frequently applied, that the patients at length recovered. Another eminent physician informed Dr. Gower, that he had met with four instances of a similar kind.

"I have already conversed (says Mr. Ring) with some of the most eminent physicians of this metropolis, on the subject of Dr. Kinglake's plan; but have not yet been able to find one bold enough to adopt the practice."

Mr. Thomas seems not unfavourable to the cooling treatment in gout, but confesses that he seldom adopts it to its fullest extent, except in young and vigorous subjects. Mr. Wadd and Mr. Leese are also favourable to Dr. Kinglake's practice.

The late Sir Michael Le Fleming, whenever he was attacked with gout, used constantly to put his feet into cold water before he went to bed; this he practised the night before his decease, which took place suddenly at the Admiralty.

Mr. Ring is informed by Mr. Hornsby, that the late Mr. Nicholson, lottery-office keeper, had recourse to Dr. Kinglake's refrigerant plan of treatment in gout, which enabled him to pursue his professional employment for some time; but the repelled gout was followed by an erysipelas, which occasioned intolerable itching day and night. In this habit of body, the accidental prick of a pen in the wrist was followed by mortification and death.

Sir W. Adams informs Mr. Ring, that a patient of his, while travelling in Germany, was attacked with the gout. Cold affusions were ordered, by which it was repelled, and settled on the hip; in consequence of which, he was lame two years.

Dr. Bain being asked his opinion of the refrigerant plan of treatment recommended by Dr. Kinglake, declared that he thought it rash.

Mr. Cordel, Surgeon, says, that about eight years ago, Dr. James Sims related a case to the Society in Bolt Court, in which a gentleman, who had plunged his foot into cold water in order to cure the gout, died the next morning. Dr. Sims forgets the circumstance, but relates the following case to Mr. Ring. Mr. W.C. a member of Parliament, had been severely afflicted for two years with pains in the bowels, which Dr. S. attributed to misplaced gout, and advised the Bath waters internally and externally. This advice being ultimately taken, the patient had a regular fit of the gout, and in a short time was restored to health, flesh, strength, and spirits.

In about two years after this, Dr. Sims met him in Piccadilly, on a cold frosty morning, walking very fast; and asking him the reason of it, he answered, that he was walking off a fit of the gout,

(a practice then much in vogue) in order that he might be able to attend to his duty in Parliament that evening. Dr. S. remonstrated in vain. The next day, the old complaint returned in his bowels. To remove this, he again tried the Bath waters, but without effect. He then went to Lisbon for change of air, but died as the ship entered the Tagus.

Dr. Hooper states, that a gentleman in the neighbourhood of Manchester Square, plunged his gouty limb into cold water, by the advice of a medical man ; and in five hours he was a corpse!

Dr. Latham and Dr. Baillie informed Mr. Ring of the unfortunate case of Mr. Baker, Surgeon, Uxbridge,* in a manner which convinced him, that they are of the same opinion with Dr. Haworth, Mr. Edlin, and all other rational, impartial, and unprejudiced persons on this occasion ; and that a worthy member of the medical profession, with a family, whose life apparently was in no particular danger, fell a sacrifice to his own temerity, and that of Dr. Kinglake." 135.

Dr. Cooke, in referring to this case, observes, "that reasoning from analogy, he should conclude the practice would prove injurious in gout." He does not employ cold himself.

Mr. Ring's Volume contains many cases, with remarks upon them, drawn from published works; but these we shall not notice. The evidence of Dr. Parry, of Bath, is in itself a host, and we have pointedly brought it forward in our review of his work. We confidently predict that the practice of Dr. Kinglake will every day lose ground, and that the same fate awaits it and the *Eau Medicinale.*

Mr. Ring also dips deeply into the disgraceful controversy between Kinglake and the surgeon-accoucheurs. But we shall not enter the lists on either side. We are sorry to see some respectable characters descend into the arena of contention on this occasion. Among others, we see Dr. Merriman submit his name for pitiful punsters to work up into wretched witticisms, that may make the idle stare, and the thoughtless laugh ! If Dr. Kinglake's propositions had been treated with the silence and contempt which they merit, they would never have excited an attention which they do not deserve. There are two ways of becoming notorious—by extreme folly, or superior talent. Time will decide by which of these Dr. Kinglake, his satellites, and his opponents, have gained the dear object of their ambition.

* 13th vol. Medical and Physical Journal.

